# Comparing Methods for Record Linkage for Public Health Action: Matching Algorithm Validation Study

**DOI:** 10.2196/15917

**Published:** 2020-04-30

**Authors:** Tigran Avoundjian, Julia C Dombrowski, Matthew R Golden, James P Hughes, Brandon L Guthrie, Janet Baseman, Mauricio Sadinle

**Affiliations:** 1 Department of Epidemiology School of Public Health University of Washington Seattle, WA United States; 2 HIV/STD Program Public Health–Seattle and King County Seattle, WA United States; 3 Division of Allergy and Infectious Diseases Department of Medicine University of Washington Seattle, WA United States; 4 Department of Biostatistics School of Public Health University of Washington Seattle, WA United States; 5 Department of Global Health School of Public Health University of Washington Seattle, WA United States

**Keywords:** medical record linkage, public health surveillance, public health practice, data management

## Abstract

**Background:**

Many public health departments use record linkage between surveillance data and external data sources to inform public health interventions. However, little guidance is available to inform these activities, and many health departments rely on deterministic algorithms that may miss many true matches. In the context of public health action, these missed matches lead to missed opportunities to deliver interventions and may exacerbate existing health inequities.

**Objective:**

This study aimed to compare the performance of record linkage algorithms commonly used in public health practice.

**Methods:**

We compared five deterministic (exact, Stenger, Ocampo 1, Ocampo 2, and Bosh) and two probabilistic record linkage algorithms (fastLink and beta record linkage [BRL]) using simulations and a real-world scenario. We simulated pairs of datasets with varying numbers of errors per record and the number of matching records between the two datasets (ie, overlap). We matched the datasets using each algorithm and calculated their recall (ie, sensitivity, the proportion of true matches identified by the algorithm) and precision (ie, positive predictive value, the proportion of matches identified by the algorithm that were true matches). We estimated the average computation time by performing a match with each algorithm 20 times while varying the size of the datasets being matched. In a real-world scenario, HIV and sexually transmitted disease surveillance data from King County, Washington, were matched to identify people living with HIV who had a syphilis diagnosis in 2017. We calculated the recall and precision of each algorithm compared with a composite standard based on the agreement in matching decisions across all the algorithms and manual review.

**Results:**

In simulations, BRL and fastLink maintained a high recall at nearly all data quality levels, while being comparable with deterministic algorithms in terms of precision. Deterministic algorithms typically failed to identify matches in scenarios with low data quality. All the deterministic algorithms had a shorter average computation time than the probabilistic algorithms. BRL had the slowest overall computation time (14 min when both datasets contained 2000 records). In the real-world scenario, BRL had the lowest trade-off between recall (309/309, 100.0%) and precision (309/312, 99.0%).

**Conclusions:**

Probabilistic record linkage algorithms maximize the number of true matches identified, reducing gaps in the coverage of interventions and maximizing the reach of public health action.

## Introduction

### Background

A central goal of public health surveillance is to provide continuous and systematically collected health-related data to inform public health practice and guide interventions to improve individual and population health [1]. For example, health departments in the United States use HIV surveillance data [2-5] to identify people living with HIV (PLWH) who are not engaged in HIV care to provide assistance and services to facilitate care engagement—a strategy known as Data to Care [6-12]. In this way, surveillance data are used to improve both HIV care and prevention as well as to reduce inequities in access and utilization of HIV care resources to improve the well-being of vulnerable populations with HIV.

When used in isolation from other sources of information, public health surveillance can be inefficient and ineffective. In the case of Data to Care, many PLWH who appear to be out of care in HIV surveillance data because they have not had a recent HIV viral load or CD4 test have actually moved out of the jurisdiction and engaged in HIV care elsewhere [4,13,14]. Thus, Data to Care strategies that rely entirely on HIV surveillance data involve time-consuming individual case investigations to determine whether persons are truly out of care, although that information is often readily available in other data sources, such as Ryan White–funded care programs, sexually transmitted disease (STD) surveillance, electronic health records, or HIV surveillance systems in other jurisdictions. The Centers for Disease Control and Prevention (CDC) is supporting efforts to match surveillance data between jurisdictions through programs such as the *black box* system, in which HIV surveillance data from multiple jurisdictions are matched to identify PLWH who have moved from one jurisdiction to another [15,16]. In addition, several health departments are seeking to improve real-time record linkage between HIV and STD surveillance to provide HIV care relinkage services as part of STD partner services [12,17].

Despite the widespread use of record linkage techniques throughout public health, little information is available to guide this process from the perspective of algorithm accuracy and the implications of missing true matches and identifying false matches. There are two primary approaches to record linkage: deterministic algorithms and probabilistic algorithms [18-20]. Deterministic algorithms use exact matching on specific variables or a set of matching rules to identify matched record pairs [18]. In contrast, probabilistic algorithms use statistical methods to identify the optimal set of matches, which often involves estimating and thresholding the probability that two records are a match [18,21,22]. Probabilistic algorithms typically have higher recall than deterministic algorithms, especially when linking databases that have high rates of data quality errors [23,24]. However, probabilistic algorithms also tend to be more computationally complex than deterministic algorithms and may require more computing resources to implement in practice [18,20].

Recent studies of record linkage involving health department HIV/STD surveillance data have presented deterministic algorithms to link HIV surveillance data with other data sources, improve the quality of HIV surveillance data, and facilitate Data to Care investigations [16,25]. These algorithms are enticing because they are not computationally complex and can be executed quickly [18-20]. As they are rule based, deterministic algorithms are intuitive to understand, easy to implement, and easy to modify. In addition (and perhaps more importantly), deterministic algorithms typically have low rates of false-positive matches. As a major concern of working with HIV data is inadvertent disclosure of HIV status, minimizing false matches is crucial to preserving individual privacy. However, although deterministic algorithms may be highly specific, they may be overly conservative in identifying matches, leading to large numbers of missed matches. Missed matches represent missed opportunities to deliver public health interventions to individuals who need them, and depending on their population distribution, missed matches could magnify health inequities. Probabilistic algorithms could potentially offer increased sensitivity compared with deterministic algorithms, while still identifying a small number of false matches.

### Objectives

The performance of deterministic algorithms compared with probabilistic algorithms in the context of public health record linkage is unknown. The goal of this study was to compare the recall, precision, and computation time of record linkage algorithms often used in HIV/STD programs to better define the trade-offs between these algorithms in a variety of record linkage scenarios.

## Methods

### Study Design

We compared deterministic and probabilistic record linkage algorithms using two approaches. First, we compared the recall, precision, and computation time of different algorithms using paired simulated datasets, varying the quality of the data and overlap between datasets (ie, the proportion of true matches in each pair of datasets). Second, we conducted a *real-world* matching scenario involving public health surveillance data from Public Health—Seattle & King County (PHSKC) to assess whether our simulation findings were generalizable to record linkage involving real datasets, where the exact error rate and overlap are difficult to assess.

This study received a human subjects research exemption from the University of Washington Institutional Review Board because it involves the use of simulated data and public health surveillance data used to inform and improve existing operational public health department activities.

### Matching Algorithms

We compared seven algorithms used to conduct record linkage involving public health surveillance data: exact matching, four deterministic, and two probabilistic algorithms (Table 1). The exact matching algorithm identifies the matched pairs of records between two datasets using an exact match on first name, last name, and year of birth. This was chosen as a *base case* algorithm because it uses the simplest rule set to match two datasets. The four deterministic algorithms (*Stenger*, *Ocampo 1*, *Ocampo 2*, and *Bosh*) define rule sets for identifying a match using patient-identifying information, such as first name, last name, date of birth, gender, and race (Table 1) [16,25]. The Ocampo and Bosh algorithms also include matching criteria that require social security numbers (SSNs), which were omitted from our study because we did not have SSNs in the datasets used. In addition, the original Ocampo and Bosh algorithms used sex at birth, whereas we have used current gender. These modifications to these algorithms are noted in Table 1. These algorithms were chosen because they have been recently cited as matching algorithms used to conduct record linkage involving HIV surveillance data. Notably, the Ocampo algorithms have been used by the CDC to match interstate HIV surveillance data [15]. The Stenger algorithm was obtained directly from the PHSKC HIV/STD program, where it has been implemented for several record linkage projects involving HIV surveillance data. This algorithm was also recently used by the Mississippi State Department of Health to link their HIV and STD surveillance databases to integrate HIV care relinkage services into STD partner services [17].

The two probabilistic algorithms are *fastLink* and *beta record linkage* (BRL). fastLink is an implementation of the traditional Fellegi-Sunter approach to record linkage [21,26]. This approach uses comparisons of the shared fields between two datasets (ie, first name, last name, year of birth, month of birth, day of birth, gender, and race) to compute the conditional probability that each record pair is a match. Record pairs are classified as *matches* or *nonmatches* based on thresholding these conditional probabilities. BRL is similar to the Fellegi-Sunter approach but uses a Bayesian implementation to explore the space of plausible matching configurations between the datafiles [22]. By using a Bayesian approach, BRL allows for quantifying uncertainty on the matching decisions and finds the optimal set of matches by minimizing the expected misclassification errors based on a loss function.

**Table 1:** Record linkage algorithms.

Algorithm	Match criteria	Source
Exact match	Exact match on first name, last name, AND year of birth	Not applicable
Stenger	Best record pairs with a score of 50+ based on the following criteria: +20 points: first 3 letters of the last name and 2 letters of the first name +15 points: exact match on the full name +15 points: match on birth year (±2 years) +5 points: exact match on the year of birth +10 points: exact match on the month of birth +5 points: exact match on the day of birth	Public Health Seattle King County and Avoundjian et al [17]
Ocampo 1	Record pairs that met the following criteria: Exact^a^: last name, first name, date of birth, race, gender^b^, AND SSN^c^ OR Very high^a^: (last name, first name, date of birth, AND gender^b^) OR SSN OR High: last name, first name, date of birth, AND (gender^b^ OR race)	Ocampo et al [16]
Ocampo 2	Record pairs that matched in Ocampo 1 OR met the following criteria: Medium high: last name, first name (Soundex), date of birth, or gender^b^	Ocampo et al [16]
Bosh	Records that met any of the following matching keys: Full last name+first 6 letters of first name+full date of birth First letter of the last name+letters 3 to 10 of the last name+letters 2 to 9 of the first name+full date of birth Letters 2 to 7 of the last name+first 6 letters of the last name+full date of birth First 2 letters of the last name+first 3 letters of the first name+full SSN+full date of birth^d^ Full last name+first 3 letters of the first name+full date of birth Letters 3 to 5 of the last name+first 3 letters of the first name+full date of birth First 4 letters of the last name+first 4 letters of the first name+full date of birth First letter of the last name+letters 3 to 10 of the last name+letters 2 to 9 of the first name+month and year of birth^e^ First letter of the last name+letters 3 to 10 of the last name+letters 2 to 9 of the first name+day and year of birth^e^ Full SSN^d,e^ First 5 letters of the last name+first 4 letters of the first name+month and year of birth^e^ First letter of the last name+letters 3 to 10 of the last name+letters 2 to 9 of the first name+(day OR month of birth)+year of birth, switching the first and last names in 1 dataset^e^ First 5 letters of the last name+first 4 letters of the first name+month and year of birth, switching the first and last names in 1 dataset^e^	Bosh et al [25]
fastLink (Fellegi-Sunter)	Calculates match/nonmatch weights using an expectation maximization algorithm and computes a match probability for each record pair. Pairs are classified as a match if their match probability is above 0.85. The following fields are used to estimate the match probability: First name and last name: partial match using Jaro Winkler string distance, with 3 agreement levels^f^ Year of birth, month of birth, day of birth, gender and race: exact match	Enamorado et al [26]
Beta Record Linkage	Uses a Gibbs sampler to sample plausible matching configurations and uses a loss function to identify the optimal set of matching pairs. The following fields are used by the algorithm: First name and last name: partial match using Levenshtein string distance, with 4 agreement levels^g^ Year of birth, month of birth, day of birth, gender, and race: exact match	Sadinle [22]

^a^We omitted social security number from the exact and very high match tiers because of lack of social security number data.

^b^Original algorithm used birth sex instead of gender.

^c^SSN: social security number.

^d^Key was not implemented because of lack of social security number data.

^e^These keys require the following additional criteria to be met to be considered a match: exact match on gender OR full date of birth AND first name in the HIV dataset not among the 20 most common names in the HIV dataset AND last name in the HIV dataset not among the 20 most common names in the HIV dataset. Note: the original algorithm used birth sex instead of gender in these criteria. In addition, the original criteria also required a match on digits 1 to 4 and 6 to 9 of social security number, which was not implemented because of lack of social security number data.

^f^FastLink’s default agreement levels for partially matched fields: 0 to 0.87: not a match, 0.88 to 0.91: partial match, and 0.92+: exact match.

^g^Beta record linkage’s default agreement levels for partially matched fields: 0 to 0.49: not a match, 0.5 to 0.74: probable nonmatch, 0.76 to 0.998: probable match, and 0.99+: exact match.

### Hypothetical Matching Scenario

To compare record linkage algorithm performance in the context of public health action, we considered the scenario of linking records between HIV and STD surveillance data to identify syphilis cases reported in the past year among PLWH. Such record linkage is conducted by many health departments in the United States as a way to integrate HIV care engagement activities into syphilis partner services. We assumed that both HIV and STD surveillance data contain the following shared fields that can be used for record linkage: first name, last name, date of birth (year, month, and day), gender, and race.

### Simulation Study

Simulations were used to compare the accuracy of the selected record linkage algorithms in scenarios with varying dataset size, overlap, and measurement error. GeCo (Australia National University, Canberra, Australia), a Python-based program that creates realistic datasets of personal information, was used to generate pairs of datasets based on STD surveillance data from PHSKC’s partner services data system, known as Public Health Information Management System (PHIMS) [27]. In each simulation, we generated two datasets containing records of 2000 individuals each. A number of individuals were included in both datasets, which we refer to as the *overlap* between the datasets. We considered scenarios where 5%, 10%, 25%, and 50% of individuals overlapped. To generate each pair of datasets, we used the distribution of values for each field from PHIMS. Using PHIMS, we created frequency tables for first and last names, year of birth, gender (male, female, transgender male, and transgender female), and race/ethnicity (Asian, black, Hispanic/Latinx, Native American/Alaska Native, Native Hawaiian/other Pacific Islander, white, other, and multiple race). We created a joint frequency table for month and day of birth, giving an equal sampling weight for each day of the year. For each individual, a value was sampled from each frequency table to generate a number of clean records, which were then *corrupted* to create the datasets. For each pair of datasets, the first dataset consisted of *clean* records, and the second dataset consisted of *corrupted* records. Each corrupted record has a fixed number of erroneous fields that are selected at random. For each dataset size and overlap scenario, we generated datasets containing 1, 2, 3, 4, and 5 erroneous fields per record. The types of errors introduced into each field were selected at random from a set of possibilities that vary from field to field (Multimedia Appendix 1). The types of errors are edits (insertions, deletions, substitutions, and transpositions of characters in a string), keyboard (typing errors based on a QWERTY keyboard layout), phonetic (using a list of predefined phonetic rules), value swap (an entire value is swapped with another value selected from a predefined list of possible values), and missing values. The probability of missing values was determined by the frequency of missing values for each field in PHSKC’s STD surveillance data. The probabilities of the remaining error types were defined based on the default probabilities provided by GeCo.

We matched each pair of datasets using each record linkage algorithm. After simulated data were created, we did not further modify the data (eg, modifying date values with missing date parts) before inputting them into any of the algorithms. We measured each algorithm’s *recall* (ie, sensitivity, the proportion of true matches identified by the algorithm) and *precision* (ie, positive predictive value, the proportion of algorithm matches that were true matches). Each matching scenario was simulated 100 times, and we calculated the mean and standard deviation of recall and precision for each algorithm across these replicates. In addition, we measured the computational performance of each algorithm in terms of their average runtime. We ran each matching algorithm 20 times while fixing the overlap between the two datasets (50% of the individuals in the second dataset overlap with those in the first dataset) and the number of erroneous fields (one erroneous field per record) and varying the size of the second dataset (10%, 25%, 50%, and 100% of the first dataset). We then calculated the mean and standard deviation of computation time for each algorithm.

### Real-World Matching Scenario

In our *real-world* matching scenario, we linked PHSKC HIV (Electronic HIV/AIDS Reporting System [eHARS]) and STD (PHIMS) surveillance data to identify PLWH who had a syphilis diagnosis in 2017. In 2017, there were 885 case-patients with a syphilis infection reported in King County. There were 17,415 PLWH in eHARS, which includes all persons living with diagnosed HIV in Washington state. As there is no shared unique identifier between PHIMS and eHARS, we did not have a gold standard against which we could compare each matching algorithm’s performance. Thus, we defined true matches and true nonmatches using a composite of the matching decisions made by each of the algorithms (*composite standard*). If all the algorithms identified a pair of records as a match, we considered it a true match. If none of the algorithms identified a pair of records as a match, it was considered a true nonmatch. When there was a lack of consensus between the record pairs, we manually reviewed the records to determine whether they were a true match or nonmatch. As in the simulations described above, we made no modifications to any date values with missing date parts before inputting them into the algorithms (<0.1% of records had missing date parts). We calculated the precision and recall of each algorithm. In addition, we measured the *value and error added* by each algorithm beyond exact matching, which we considered as the baseline algorithm. We measured *value added* as the number of additional true matches and *error added* as the additional false matches identified by each algorithm over and beyond exact matching.

Dataset generation and corruption were done using GeCo and Python 2.7. All other analyses were done using R version 3.5.2. Python and R programs used to perform simulations, perform the real-world match, and measure computational performance are provided as supplemental material (Multimedia Appendix 2).

## Results

### Simulations

**Figure 1:**
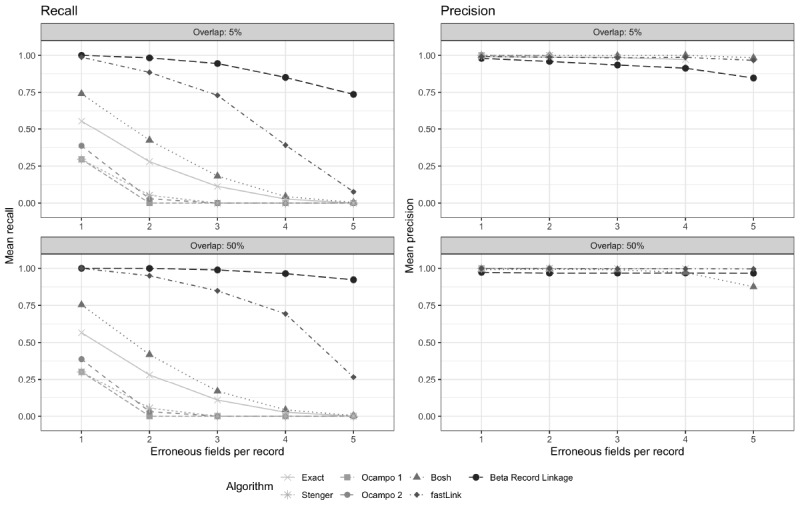
Simulations: record linkage algorithm recall/precision.

The selected deterministic algorithms had a lower recall than the selected probabilistic algorithms, regardless of the overlap or the number of erroneous fields per record (Figure 1 and Multimedia Appendix 1). The exact algorithm had a recall of between 56% (5% overlap) and 57% (50% overlap) when there was one erroneous field per record, and its recall decreased as the number of erroneous fields per record increased. The exact matching algorithm’s precision was between 99% and 100% when there were three or fewer erroneous fields per record (Multimedia Appendix 1). The Stenger, Ocampo 1, and Ocampo 2 algorithms had similar recall and precision but had lower recall than the exact match. When there was only one erroneous field, both the Stenger and Ocampo 1 algorithms had a recall of 30%, whereas the Ocampo 2 algorithm had a recall of 39%, regardless of the dataset size and overlap. The precision for all three algorithms was 100% when there was only one erroneous field per record. All three algorithms failed to identify any matches when there were at least three erroneous fields. The Bosh algorithm had the highest recall of the five deterministic algorithms. When there was one erroneous field per record, the Bosh algorithm’s recall ranged between 74% (5% overlap) and 75% (50% overlap). However, its recall decreased to less than 20% in scenarios with at least three erroneous fields per record. The precision for the Bosh algorithm was high across all scenarios (between 88% and 100%).

fastLink and BRL had better recall than the deterministic algorithms. In the one erroneous field per record scenario, both fastLink and BRL had about 100% recall, regardless of the dataset overlap. In the three erroneous field scenario, fastLink’s recall ranged between 73% (5% overlap) and 85% (50% overlap), whereas BRL’s recall ranged between 94% and 99%. In the five erroneous field scenario, fastLink’s recall was between 8% and 27%, whereas BRL’s recall was between 74% and 92%. The precision of both algorithms was high across all scenarios (fastLink: 97%-100% and BRL: 85%-100%).

### Computational Performance

**Figure 2:**
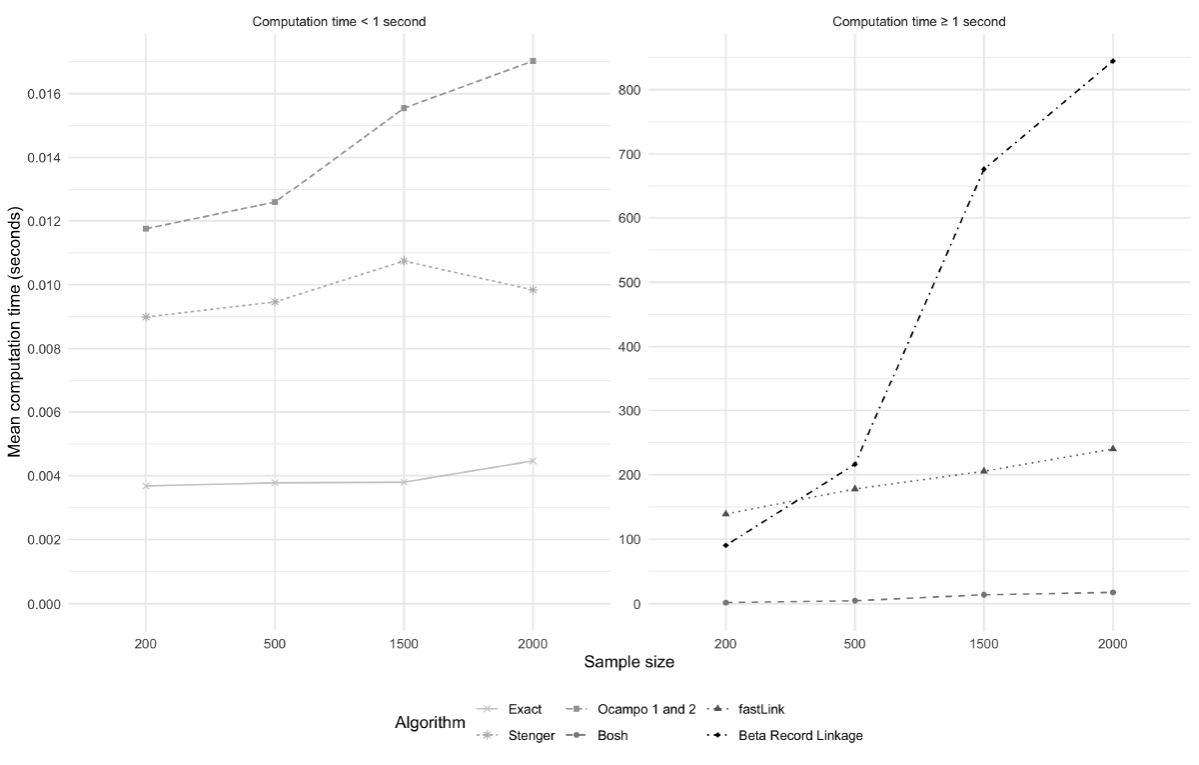
Record linkage algorithm matching computational performance. Average computational time after 20 replications in scenario where overlap (50%) and number of erroneous fields per record (1) were fixed and size of second dataset was varied (10%, 25%, 50%, and 75% of first dataset [N=2000]).

The exact, Ocampo, and Stenger algorithms took an average of about 0.01 seconds to compute, even when the datasets being compared contained 2000 records (Figure 2). The Bosh algorithm took between 2 seconds and 18 seconds to compute, depending on the dataset size. The two probabilistic algorithms took a longer time to compute than all the deterministic algorithms. fastLink took an average of between 2.3 min and 4 min to compute. On average, BRL performed faster than fastLink when the second dataset contained 200 records (1.5 min vs 2.3 min) but was the slowest algorithm in every other scenario. BRL, on average, took between 3.6 min (second dataset N=500) and 14.1 min (second dataset N=2000) in the remaining scenarios.

### Real-World Matching Scenario

Among the 885 case-patients with any syphilis infection in King County in 2017, a majority (760/885, 85.8%) were men who have sex with men (MSM). Nearly half of the patients were white (436/885, 49.3%), 12.8% (113/885) were black, and 20.5% (182/885) were Hispanic/Latinx. Among the 17,415 PLWH in PHSKC’s eHARS database, 14,887 (85.48%) were male (12,640/17,415, 72.58% MSM), 10,293 (59.10%) were white, 2965 (17.10%) were black, and 2376 (13.67%) were Hispanic/Latinx.

There were 367 record pairs classified as a match by any of the algorithms. Of these, the algorithms disagreed on 113 record pairs, which were manually reviewed to determine their true match status. According to our composite standard, there were 309 true matches, representing 35% of all case-patients with a syphilis infection in 2017 and 1.8% of all PLWH in eHARS. The exact matching algorithm identified 256 true matches and one mismatch (Multimedia Appendix 3). Compared with this algorithm, the Stenger and Ocampo 1 algorithms identified two fewer true matches and did not have any mismatches. The Ocampo 2 algorithm identified three more matches than the exact matching algorithm and also had no mismatches. The Bosh algorithm identified 36 additional true matches but also identified 20 additional false matches. Both fastLink and BRL identified 53 additional true matches. However, fastLink had 33 additional false matches, whereas BRL only had two additional false matches.

**Figure 3:**
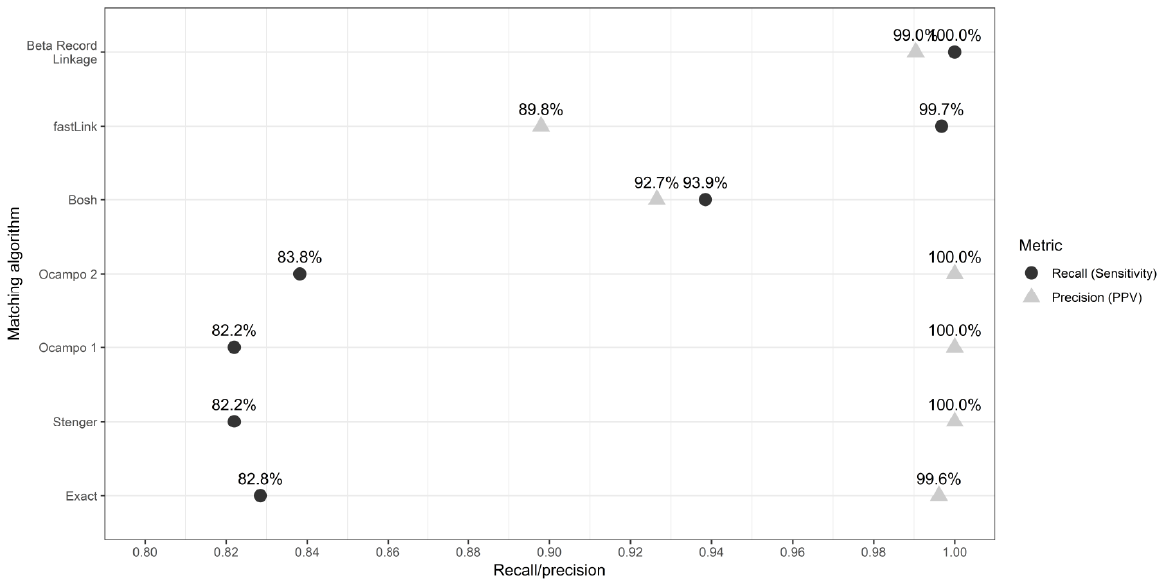
Real-world matching scenario: record linkage algorithm recall and precision. PPV: positive predictive value.

Compared with our composite standard, all the deterministic algorithms had lower recall than the probabilistic algorithms (Figure 3). The recall of the exact, Stenger, Ocampo 1, and Ocampo 2 algorithms ranged between 82% and 84%. The recall of the Bosh algorithm was about 94%, and the recall of fastLink and BRL was 100%. The precision of the deterministic algorithms (except for Bosh) was overall higher than the precision of the probabilistic algorithms. The Stenger, Ocampo 1, and Ocampo 2 algorithms had 100% precision, whereas the exact algorithm had 99.6% precision. The precision of the Bosh algorithm was about 93%, and the precision of fastLink was about 90%. BRL had a precision of 99%, which was the lowest trade-off between recall and precision.

## Discussion

### Principal Findings

Using simulations, we found that the probabilistic algorithms we evaluated had substantially better recall than the selected deterministic algorithms, while the deterministic algorithms had higher precision. However, in scenarios with three or more erroneous fields per record, nearly all the deterministic algorithms (except the Bosh algorithm) failed to identify any matches, which diminishes their utility in record linkage scenarios where data quality is poor. In contrast, both BRL and fastLink offered high recall without sacrificing much in terms of precision. In addition, in a *real-world* comparison, BRL had the highest recall with only a minimal sacrifice in precision and was the best performing algorithm overall.

Our findings suggest that although deterministic algorithms offer a high degree of precision, they are highly sensitive to data quality issues and may miss a substantial number of matches even in situations where there is only one erroneous field per record. The recall of deterministic algorithms can be improved by implementing more matching rules (as in the case of the Bosh algorithm [25]), but this also results in lower precision. Furthermore, even with additional match keys, deterministic algorithms still do not reach the level of recall offered by probabilistic algorithms.

Surprisingly, the Bosh and fastLink algorithms had low precision in our real-world match, despite having very high precision in simulations. For fastLink, this may be a limitation of the algorithm, which tends to lose precision in situations where the overlap between datasets is small or there is a large difference in the size of the datasets being linked [26]. The lack of SSN may have led to the Bosh algorithm’s lower precision in the real-world match. The false matches identified by the Bosh algorithm were identified because they met matching keys 8 to 14, which require additional criteria to be considered a match (Table 1). As noted in the original Bosh article, these additional criteria were added to reduce possible false matches. Although we implemented most of the additional criteria, they include a partial match on SSN (ie, match on digits 1-4 and 6-9 of SSN), which was omitted from this study. If SSN was included, we may have eliminated the false matches identified by the less strict matching keys, resulting in a higher observed precision for this algorithm.

### Public Health Implications

In the context of public health action, choosing a record linkage algorithm that prioritizes the identification of true matches is critical to preventing gaps in the provision of public health interventions to those who are most in need of assistance. Choosing overly conservative record linkage algorithms that prioritize precision over recall could increase gaps among these groups in public health prevention delivery and may amplify disparities among marginalized populations. Previous studies have demonstrated that imperfect record linkage algorithms may disproportionately miss women, older individuals, and persons of minoritized races/ethnicities and lower socioeconomic status [28-31]. The use of probabilistic record linkage methods (such as BRL and fastLink) or more complex deterministic algorithms (such as the Bosh algorithm) would result in a large increase in the reach of public health interventions relying on the linkage of data systems, which offsets small decreases in match precision.

A disadvantage of probabilistic algorithms is their computational complexity. While the computational time of the deterministic algorithms is generally under 1 second, both probabilistic methods took minutes to compute. For applications that require near-instant record linkage of large databases, probabilistic algorithms may not be practical because of their slow computation time; however, such applications may be relatively uncommon in practice. When record linkage is done on a daily or less frequent basis, the increased computation time of fastLink and BRL is less problematic. Importantly, fastLink was designed to outperform other approaches to probabilistic record linkage algorithms when datasets are very large [26]. In these situations, fastLink may have even greater gains compared with slower methods such as BRL, although it may still be slower than deterministic algorithms. In addition, because of their increased computational complexity, BRL and fastLink require more memory and processing power than the deterministic algorithms. Both BRL and fastLink required over 4 GB of RAM and a 64-bit version of R, which may be a limitation of using these algorithms in resource-limited settings. However, 64-bit computing and 4 or more GB of RAM are becoming increasingly common, suggesting that these barriers would be less problematic in the future. As of May 2019, the estimated minimum cost of a new business desktop with these specifications is about US $400.

Another advantage of deterministic algorithms is that these are easier to implement in different programming languages. Matching rules used by the deterministic algorithms we evaluated are relatively intuitive and translatable to multiple programming languages. Although fastLink has thorough documentation and support, modifications to the algorithm require an understanding of the Fellegi-Sunter record linkage methodology and the R programming language [26]. Modifications to BRL are particularly challenging, as there is currently limited documentation on the method [22]. In addition, much of the BRL algorithm is implemented in the C programming language, an additional prerequisite to making modifications to the algorithm. To address these barriers, we have provided R programs for each algorithm in a *Load, Clean, Func, Do* framework, a portable and flexible organizational structure for developing R projects, to implement them in practice (Multimedia Appendix 2) [32].

### Limitations

Our study has several limitations. First, in our simulations, we assumed a uniform error rate across all records in each matching scenario. As our probabilistic algorithms use information from all records, this may have misrepresented how well they perform when linking datasets that contain a wide range of erroneous fields per record, including records that have 0 erroneous fields. Indeed, in our real-world match scenario, in which record quality was more variable, BRL had much higher precision than in our simulations, suggesting that it is able to leverage information from record pairs that have high data quality to make decisions about record pairs that have poor data quality.

Second, both the Bosh and Ocampo algorithms include matching keys that involve SSN, which is not available in PHSKC’s STD surveillance database. This may have resulted in an underestimation of the performance of these algorithms. In the Bosh algorithm, SSN is used as additional criteria to reduce mismatches for matching keys that are very broad, and its inclusion may have resulted in improved precision. In the original Bosh study, 1.7% of true matches were identified using SSN alone, suggesting that if SSN was available, we would have observed a very slightly improved recall of the Bosh algorithm, although it probably would not have reached the levels of recall observed with the probabilistic algorithms [25]. In addition, if SSN had been available, it could have also been included in both probabilistic algorithms, which could have possibly improved their recall and precision as well.

Third, we have only considered deterministic and probabilistic algorithms that can be implemented in R and have excluded algorithms that require third-party software (eg, the Link King and CDC’s Link Plus) and novel record linkage methodologies (eg, active, supervised, and unsupervised learning algorithms). Third-party software for record linkage offers a point-and-click interface for implementing probabilistic (and deterministic) record linkage methodologies. Both the Link King and Link Plus, two popular applications for conducting record linkage involving public health surveillance databases, use the Fellegi-Sunter methodology for conducting probabilistic record linkage, which is the same methodology used by fastLink. Supervised learning–based and active learning–based algorithms may yield greater match quality than probabilistic or deterministic algorithms in cases where databases are to be linked prospectively or when training data are available (in the case of supervised learning) [19]. These algorithms use data on record pairs that are known to be matches or nonmatches to develop a predictive model that is used to classify record pairs in the databases that are being linked as matches or nonmatches. As these algorithms require a training dataset of known matches and nonmatches (something neither the probabilistic nor the deterministic algorithms we evaluated required), we chose to exclude them from our analysis. Further research is needed to assess the performance and utility of these techniques in conducting record linkage for public health action as well as the feasibility of implementing them in practice.

Finally, for the probabilistic matching algorithms we evaluated, we only considered their default parameterizations. We chose to evaluate these algorithms using their default (or *out-of-the-box*) implementations, as this would represent a baseline level of their performance. Modifying the parameters for fastLink and BRL, such as the string distance measure used to match string variables or the number of partial agreement levels, could improve their performance. Importantly, fastLink and BRL use different default methods to match string variables (eg, first name and last name). This may partially explain why BRL had better recall than fastLink in our simulations and a lower trade-off between recall and precision in our real-world match. In addition, the use of a blocking scheme, such as grouping record pairs on the first two letters of the first name before they are compared by the algorithm, may have improved both the precision and computational performance of these algorithms. Future studies should consider evaluating the use of blocking on algorithm performance in the public health practice setting.

### Conclusions

In conclusion, public health interventions that involve record linkage of multiple data systems should carefully consider their choice of record linkage algorithm. This choice should be based not only on reducing false matches but also on maximizing intervention coverage. Record linkage methodologies that do not seek to maximize true matches, especially in the context of imperfect data quality, limit the reach of public health interventions and could exacerbate existing health disparities. Probabilistic algorithms, such as BRL, can maximize the number of true matches identified without sacrificing precision and should be considered as the first choice when using record linkage for public health action.

## Appendix

Multimedia Appendix 1 Additional details about data generation and tables describing simulation results.

Multimedia Appendix 2 Python and R programs used to conduct simulations and real-world match.

Multimedia Appendix 3 Real-world matching scenario: value and error added over exact matching algorithm.

## Abbreviations

BRL: beta record linkage
CDC: Centers for Disease Control and Prevention
eHARS: Electronic HIV/AIDS Reporting System
MSM: men who have sex with men
PHIMS: Public Health Information Management System
PHSKC: Public Health—Seattle & King County
PLWH: people living with HIV
SSN: social security number
STD: sexually transmitted disease
