# Supplementary material for: Comparing Methods for Record Linkage for Public Health Action: Matching Algorithm Validation Study
Source: JMIR Public Health Surveill. 2020 Apr 30;6(2):e15917. doi: 10.2196/15917 (PMC7226047; doi:10.2196/15917)
Supplement: Multimedia Appendix 1 [file publichealth_v6i2e15917_app1.docx]

## Multimedia Appendix 1: Supplemental Tables

Table S1. Error types and probabilities for simulated datasets

| **Field** | **Error Type^1^** | **Probability** |
| --- | --- | --- |
|  |  |  |
| First name | Edits | 9.9% |
|  | Keyboard | 9.9% |
|  | Phonetic | 69.3% |
|  | Value swap | 9.9% |
|  | Missing | 1% |
| Last name | Edits | 14.55% |
|  | Keyboard | 14.55% |
|  | Phonetic | 67.9% |
|  | Missing | 3% |
| Year, month, and day of birth | Edits^2^ | 99.93% |
|  | Missing | 0.07% |
| Gender, race/ethnicity | Value swap^3^ | 99.93% |
|  | Missing | 0.07% |
| ^1^Error types: edits – insertion, deletion, substitution, or transposition of characters in a string; keyboard – typing errors based on QWERTY keyboard layout (values close to each other on a QWERTY keyboard more likely to be swapped than values farther apart); phonetic – character substitutions based on list of phonetically similar characters; value swap – swaps first name for a nickname, alternate spelling or alias based on a pre-defined list of values; missing – value swapped with blank value to represent missing data  ^2^Edit errors restricted to substitution/transposition of numeric characters only  ^3^Values swapped based on list of valid genders, race/ethnicities defined in frequency tables | | |

|  |  | **Record Linkage Algorithm** | | | | | | |
| --- | --- | --- | --- | --- | --- | --- | --- | --- |
| **Erroneous fields^2^** | **Overlap^3^** | **Exact** | **Stenger** | **Ocampo 1** | **Ocampo 2** | **Bosh** | **FastLink** | **Beta Record Linkage** |
|  |  |  |  |  |  |  |  |  |
| 1 | 5% | 0.56 (0.05) | 0.30 (0.05) | 0.30 (0.05) | 0.39 (0.05) | 0.74 (0.04) | 0.99 (0.02) | 1.00 (0) |
|  | 10% | 0.57 (0.03) | 0.3 (0.03) | 0.3 (0.03) | 0.39 (0.03) | 0.74 (0.03) | 1.00 (0.001) | 1.00 (0) |
|  | 25% | 0.56 (0.02) | 0.3 (0.02) | 0.3 (0.02) | 0.39 (0.02) | 0.74 (0.02) | 1.00 (0.0002) | 1.00 (0) |
|  | 50% | 0.57 (0.02) | 0.30 (0.01) | 0.30 (0.01) | 0.39 (0.02) | 0.75 (0.02) | 1.00 (0.0001) | 1.00 (0.0001) |
| 3 | 5% | 0.11 (0.03) | 0 (0) | 0 (0) | 0 (0) | 0.18 (0.03) | 0.73 (0.05) | 0.94 (0.02) |
|  | 10% | 0.11 (0.02) | 0 (0) | 0 (0) | 0 (0) | 0.18 (0.02) | 0.76 (0.04) | 0.96 (0.01) |
|  | 25% | 0.11 (0.01) | 0 (0) | 0 (0) | 0 (0) | 0.17 (0.02) | 0.81 (0.02) | 0.97 (0.01) |
|  | 50% | 0.11 (0.01) | 0 (0) | 0 (0) | 0 (0) | 0.17 (0.01) | 0.85 (0.01) | 0.99 (0.003) |
| 5 | 5% | 0 (0) | 0 (0) | 0 (0) | 0 (0) | 0.01 (0.01) | 0.08 (0.04) | 0.74 (0.05) |
|  | 10% | 0 (0) | 0 (0) | 0 (0) | 0 (0) | 0.005 (0.005) | 0.14 (0.04) | 0.82 (0.04) |
|  | 25% | 0 (0) | 0 (0) | 0 (0) | 0 (0) | 0.004 (0.003) | 0.22 (0.02) | 0.89 (0.01) |
|  | 50% | 0 (0) | 0 (0) | 0 (0) | 0 (0) | 0.01 (0.002) | 0.27 (0.02) | 0.92 (0.01) |
| ^1^Dataset size fixed to 2000 records for both datasets  ^2^Number of erroneous fields per record  ^3^Percent of records in second dataset that had a match in first dataset | | | | | | | | |

Table S2. Simulations: record linkage algorithm recall (mean (SD))^1^

|  |  | **Record Linkage Algorithm** | | | | | | |
| --- | --- | --- | --- | --- | --- | --- | --- | --- |
| **Erroneous fields^2^** | **Overlap^3^** | **Exact** | **Stenger** | **Ocampo 1** | **Ocampo 2** | **Bosh** | **FastLink** | **Beta Record Linkage** |
|  |  |  |  |  |  |  |  |  |
| 1 | 5% | 0.99 (0.01) | 1.00 (0) | 1.00 (0) | 1.00 (0.003) | 1.00 (0.004) | 0.99 (0.01) | 0.98 (0.02) |
|  | 10% | 1.00 (0.01) | 1.00 (0) | 1.00 (0) | 1 (0.001) | 1 (0.0015) | 1 (0.004) | 0.99 (0.009) |
|  | 25% | 1.00 (0.002) | 1.00 (0) | 1.00 (0) | 1 (0.001) | 1 (0.0035) | 1 (0.002) | 0.98 (0.007) |
|  | 50% | 1.00 (0.001) | 1.00 (0) | 1.00 (0) | 1.00 (0.0003) | 0.99 (0.01) | 1.00 (0.001) | 0.97 (0.01) |
| 3 | 5% | 0.99 (0.04) | - | - | - | 1.00 (0.01) | 0.98 (0.02) | 0.93 (0.03) |
|  | 10% | 0.99 (0.04) | - | - | - | 1.00 (0.01) | 0.98 (0.02) | 0.93 (0.028) |
|  | 25% | 0.99 (0.02) | - | - | - | 1.00 (0.01) | 0.99 (0.01) | 0.95 (0.018) |
|  | 50% | 1.00 (0.004) | - | - | - | 0.99 (0.01) | 1.00 (0.002) | 0.97 (0.01) |
| 5 | 5% | - | - | - | - | 0.98 (0.09) | 0.97 (0.07) | 0.85 (0.04) |
|  | 10% | - | - | - | - | 0.99 (0.04) | 0.98 (0.03) | 0.89 (0.03) |
|  | 25% | - | - | - | - | 0.93 (0.14) | 0.99 (0.01) | 0.95 (0.01) |
|  | 50% | - | - | - | - | 0.88 (0.16) | 1.00 (0.01) | 0.97 (0.01) |
| ^1^Dataset size fixed to 2000 records for both datasets  ^2^Number of erroneous fields per record  ^3^Percent of records in second dataset that had a match in first dataset | | | | | | | | |

Table S3. Simulations: record linkage algorithm precision (mean (SD))^1^
