# Supplementary material for: Comparing Methods for Record Linkage for Public Health Action: Matching Algorithm Validation Study
Source: JMIR Public Health Surveill. 2020 Apr 30;6(2):e15917. doi: 10.2196/15917 (PMC7226047; doi:10.2196/15917)
Supplement: Multimedia Appendix 2 [file publichealth_v6i2e15917_app2.docx]

# Multimedia Appendix 2: Simulation and real-world match programs

All programs used in the study are available as a .zip file on Open Science Framework using the following link: <https://osf.io/2jyng/?view_only=eb04dc56ae8942b09bac6125864d6cfd>. The zip file is organized as follows:

- **Dataset generation:** contains programs for generating and corrupting simulation datasets
  - *geco_sysarg.py:* Python script for generating/corrupting datasets
  - *Run GeCo batch.R*: batch run geco_sysarg.py to generate multiple simulation datasets
  - *lookup-files:* look up files needed for dataset generation/corruption
- **Simulations:** contains programs used to match simulation datasets using each matching algorithm we evaluated.
  - *00 - setup.R:* loads package libraries and prepares datasets for matching
  - *01a-01f:* loads matching algorithms
  - *02 -* *match validity.R*: loads functions for calculating algorithm recall and precision
  - *03 -* *tasks.R:* loads functions needed for simulations
  - *05 -* *run matching algorithms.R*: runs all simulation programs
  - *brl programs:* additional programs needed to run BRL algorithm
- **Real match:** contains programs for using matching algorithms in practice using the LCFD framework. The LCFD framework contains the following programs:
  - *load*.*R*: loads package libraries and necessary datasets
  - *clean*.*R:* functions for cleaning datasets and prepares them for matching
  - *func*.*R:* functions for performing matches and outputting final matched dataset
  - *do.R:* function calls to clean and match datasets
  - *main.R*: runs all other programs
