# Supplementary material for: Comparing Methods for Record Linkage for Public Health Action: Matching Algorithm Validation Study
Source: JMIR Public Health Surveill. 2020 Apr 30;6(2):e15917. doi: 10.2196/15917 (PMC7226047; doi:10.2196/15917)

Supplemental Figure. Real-world matching scenario: value and error added over exact matching algorithm.

The exact matching algorithm identified 256 true matches and one mismatch. Value added: additional true matches identified beyond exact matching algorithm. Error added: additional false matches added beyond exact matching algorithm.
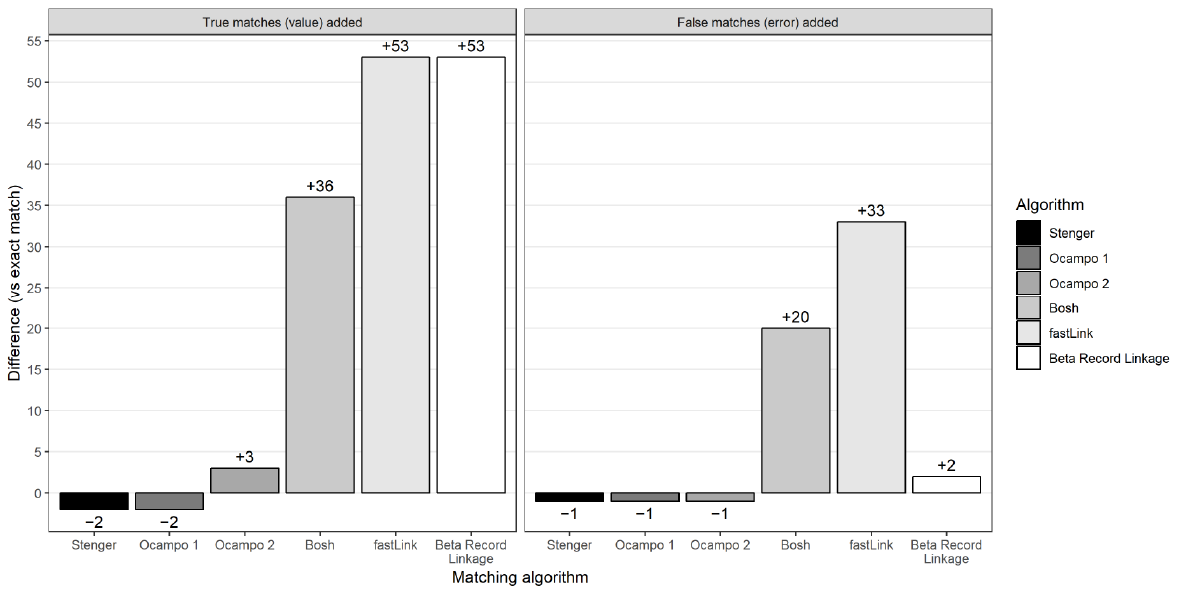

Supplement: Multimedia Appendix 3 [file publichealth_v6i2e15917_app3.docx]
